# Computational models for improving surveillance for the early detection of direct introduction of cassava brown streak disease in Nigeria

**DOI:** 10.1371/journal.pone.0304656

**Published:** 2024-08-21

**Authors:** Alex C. Ferris, Richard O. J. H. Stutt, David S. Godding, Ibrahim Umar Mohammed, Chukwuemeka K. Nkere, Angela O. Eni, Justin S. Pita, Christopher A. Gilligan

**Affiliations:** 1 Institute for Disease Modeling, Bill & Melinda Gates Foundation, Seattle, Washington, United States of America; 2 Department of Plant Sciences, University of Cambridge, Cambridge, United Kingdom; 3 Crop Science, Kebbi State University of Science and Technology, Aliero, Nigeria; 4 Central and West African Virus Epidemiology, Pôle Scientifique et d’innovation de Bingerville, Université Félix Houphoüet-Boigny, Bingerville, Côte d’Ivoire; 5 Biotechnology Department, National Root Crops Research Institute, Umudike, Nigeria; Pan African University of Life and Earth Sciences Institute, PAULESI, NIGERIA

## Abstract

Cassava is a key source of calories for smallholder farmers in sub-Saharan Africa but its role as a food security crop is threatened by the cross-continental spread of cassava brown streak disease (CBSD) that causes high yield losses. In order to mitigate the impact of CBSD, it is important to minimise the delay in first detection of CBSD after introduction to a new country or state so that interventions can be deployed more effectively. Using a computational model that combines simulations of CBSD spread at both the landscape and field scales, we model the effectiveness of different country level survey strategies in Nigeria when CBSD is directly introduced. We find that the main limitation to the rapid CBSD detection in Nigeria, using the current survey strategy, is that an insufficient number of fields are surveyed in newly infected Nigerian states, not the total number of fields surveyed across the country, nor the limitation of only surveying fields near a road. We explored different strategies for geographically selecting fields to survey and found that early and consistent CBSD detection will involve confining candidate survey fields to states where CBSD has not yet been detected and where survey locations are allocated in proportion to the density of cassava crops, detects CBSD sooner, more consistently, and when the epidemic is smaller compared with distributing surveys uniformly across Nigeria.

## Introduction

Cassava is the second largest source of calories in sub-Saharan Africa and a key food security crop for small holder farmers [1, 2]. Cassava brown streak disease (CBSD) is caused by two different vector-borne virus that are spread by the silverleaf whitefly, *Bemisia tabaci* (Gennadius 1889), and through the movement of infected planting materials [3]. Cassava brown streak virus (CBSV) and Ugandan cassava brown streak virus (UCBSV) cause chlorosis on leaves, brown streaks on the stem, and, most importantly dry, hard root necrosis that renders cassava roots inedible [4, 5]. While CBSD has been endemic in East Africa since at least the 1930s, in 2004 it began to spread westward from Uganda and has been found in Eastern Democratic Republic of the Congo but not yet in West Africa [4, 6–9].

Early detection of invasion of the disease into a new region is essential for effective management [10, 11] by providing policy makers with information to help allocate limited resources for surveillance, early detection, and response. Country-wide surveys for CBSD are currently conducted in many countries in sub-Saharan Africa using similar within-field survey protocols [12] although the total number of fields surveyed and frequency vary by country [13–28]. There are many practical limitations to how many surveys can be conducted in a single year including limited funding and availability of expert surveyors trained in identifying CBSD symptoms. Additionally, field accessibility and timing of visible foliar symptoms further limit where and when surveys can be conducted [12]. Changing the strategy for selecting fields to survey for CBSD has the potential to increase the effectiveness of detecting CBSD spread without significantly increasing the total number of fields surveyed or the logistical burden on surveyors. While there is evidence of westward spread of CBSD from Uganda through Rwanda, Zambia and the Democratic Republic of Congo [6, 8, 9, 29, 30] towards Gabon, Cameroon and Nigeria, there is also the potential for CBSD to be introduced directly for example by personal import of infected cassava planting material that is undetected at ports or borders. Here we focus on optimising surveillance strategies for early detection from direct introduction of CBSD into Nigeria, the country with the world’s largest cassava production [31]. We compare the effectiveness of different surveillance strategies for early detection of CBSD using an integrated spatially explicit epidemiological model to simulate epidemic spread at field, state, and country-wide scales.

We develop a model for country-level CBSD surveillance that integrates a landscape scale model of CBSD spread within a country with a field-level model of CBSD spread within a field [10, 32]. We generate simulated trajectories of CBSD spread across Nigeria over a 30-year period using a previously developed, spatially-explicit, landscape-scale model that takes account of cassava and whitefly abundance [32]. Individual fields are chosen for surveillance from amongst replicate simulations of landscape spread of CBSD and within these fields we model the spread of CBSD and within field surveys using a stochastic, spatially explicit, agent-based model [10].

Although we model the spread of CBSD throughout the cassava growing states in Nigeria, we have highlighted five representative states that differ in cropping density and geopolitical zone to compare the effectiveness of different surveillance strategies in detecting the potential arrival of CBSD into each state. The surveillance strategies encompass a baseline representing current practice and realistic modifications that could be made to the baseline. Specifically, we address the following questions: how does restricting fields surveyed to within 1 km of roads affect the chances of detecting incursions in states with different densities of cassava crops? How do sampling strategy and country-wide sampling effort affect the delay in detecting CBSD in a given state and the epidemic size within a state at the time of detection? We also assess the effect of using additional non-expert surveyors on epidemic size at first detection in a state.

## Methods

### Modelling CBSD spread

Theoretical infection trajectories of CBSD across Nigeria were simulated using the model developed in Godding *et al*. [32]. Briefly, this landscape scale model is a stochastic, spatio-temporal model that simulates the spread of CBSD based on whitefly and cassava density. The disease progression is tracked by dividing the landscape into a rasterized grid with cells that are approximately 1 km^2^ (depending on latitude) and simulating the percentage of cassava within the cell that is infected. The spread of CBSD is governed by a power law kernel function, where the kernel scale parameter and transmission rate were parameterised using Approximate Bayesian Computation [33, 34]. These parameters were estimated using annual disease survey data from Uganda with the first six years of data used to train the model and the subsequent six years used for its validation [6, 13, 32].

Hypothetical 30-year epidemic trajectories were generated using the landscape-scale model to simulate epidemic spread across Nigeria following the direct introduction of CBSD to a randomly selected location in Nigeria. The probability of any given starting location for direct introduction was proportional to the host density in that location. Smoothed vector density maps derived from surveys on whitefly prevalence conducted throughout Nigeria were used to characterise vector densities for input to the model [18–20]. CassavaMap [35] was used as an estimate for cassava host density within Nigeria based on production estimates by FAO and IITA while the Global Road dataset [36] was used as the source for the Nigerian road network. We focused on five representative states (Nasarawa, Plateau, Kebbi, Ogun, and Anambra) and compared times to detection and epidemic size for a set of different surveillance strategies. Additional information regarding the ethical, cultural, and scientific considerations specific to inclusivity in global research is included in the Supporting Information (S1 Checklist).

Fields to be surveyed were selected for each year of the simulation from cells containing cassava fields that were within 1 km of a road. For each epidemic trajectory, the distribution of when the individual fields in that cell became infected was constructed, and a single value was drawn from that distribution, representing the time since infection for the specific field being surveyed. The spread of CBSD within that field was then simulated using an individual-based stochastic model over one or more growing seasons. This model explicitly tracked whitefly movement between plants and the total number of plants infected in a field. For each infected plant, the model tracked the increase in viral load and the increase in foliar symptoms over time. Within-field surveys were simulated by selecting individual plants in the field and checking to see if CBSD symptoms were detected by surveyors in plants with visible symptoms. The field level model was developed and described in more detail in Ferris *et al*. [10].

### Modeling country level survey allocation strategies

Five different approaches to allocating surveys across the country were simulated in this study. In all cases, fields could only be selected for surveillance if they were within 1 km of a road to reflect the practical limitations of which fields would be accessible to surveyors [12]. The *Baseline* survey strategy reflected the current strategy used across sub-Saharan Africa in which fields are selected uniformly across a region [13–28]. The frequency and total number of fields surveyed varies by country, and in Nigeria, approximately 600 fields are surveyed annually. In practice sites are located close to roads and at approximately 10 km intervals along selected roads. In addition to the *Baseline* strategy, we implemented the following strategies:

*Single-detection*: Once CBSD infection is detected in a state, no more fields are surveyed in that state;
*Survey-adjacent*: Surveys are initially conducted in all states initially, but after CBSD infection is detected in a state, surveys are only conducted in states where CBSD infection has not yet been detected that also share a border with a CBSD-positive state;
*Host-density*: surveys are conducted in all states and the probability of selecting a given cell as a survey location is proportional to the amount of cassava plants in that cell;
*Host-density-with-single-detection*: the host-density strategy with an additional constraint that no more surveys are conducted in states after CBSD infection is detected.

For simulating within-field surveys, the same protocol was used for all five country level survey strategies. Matching the currently-used within-field survey protocol, 30 plants were surveyed per field (15 plants on two diagonal transects) [12]. There is a three-month window, from three to six months after planting, when foliar surveys can be conducted, and for simplicity we assumed that all within-field surveys were conducted six months after the start of the growing season.

We assumed that the per plant detection accuracy for CBSD based upon visual detection was 1.0 when conducted by expert surveyors. We also investigated the effect of surveying plants using image recognition via a phone app to identify CBSD, for which we assumed that the per plant detection accuracy for CBSD was 0.75, reflecting the reported true positive rate for Nuru, an android based plant disease detection app [37]. We assumed that the false positive rates for expert surveyors and for a phone app is zero. In practice, the true positive and false positive rates will vary geographically due to differences in varieties and prevalence of other cassava diseases.

To ensure that the results were representative of inherent variability of epidemic spread, captured by cross simulation variability in the landscape model, we generated 100 epidemic trajectories using the landscape-scale model where the initial site of infection was chosen randomly with a probability proportional to host density. We then evaluated the effectiveness of the different survey allocation strategies in each of the independent runs. Additionally, to take into account variability stemming from which specific fields were surveyed within a single run of the landscape-scale model, we simulated within-field survey results for a large number of fields and then subsampled from this larger pool 500 times to generate representative results.

Several metrics were used to quantify the effectiveness of the different survey allocation strategies in early CBSD detection. First, we compared the average delay in detecting epidemic spread, which is the average number of years between a given state becoming infected with CBSD and when that incursion is detected in a field. Second, the change in epidemic size at first detection in a given state was calculated as the average number of fields with infected plants at detection using one of the alternative survey allocation strategies relative to using the baseline strategy. The resulting value was then divided by the total amount of cassava plants in the state to make it easier to compare states. Finally, we compared the number of annual within-field surveys that were required to consistently detect CBSD introduction into a new state for each survey allocation strategy. To quantify this, we calculated how many annual within-field surveys were required such that in 90% of the replicate epidemic trajectories, the CBSD infection was detected within three years of introduction to a state.

### State selection

**Fig 1:**
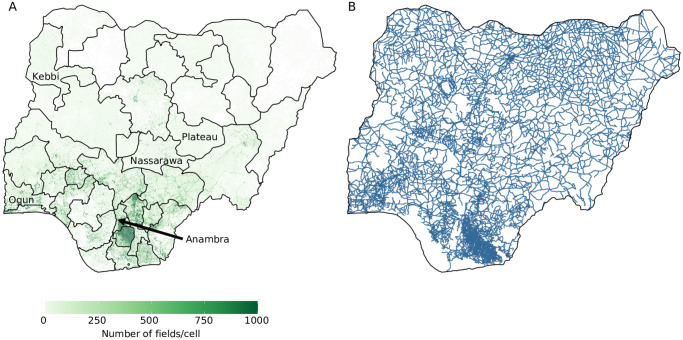
Maps of Nigeria. (A) The distribution of cassava across Nigeria with selected states used for further analysis labeled by name and (B) locations in Nigeria that are within 1 km of a road.

The effectiveness of the survey allocation methods can be expected to vary depending on the characteristics of the state. For example, the density of cassava in a state has a direct impact on the way the survey allocation strategies ‘Host-density’ and ‘Host-density-with-single-detection’ are implemented, as are other factors such as where in the states are located relative to initial sites of invasion and how quickly a state, on average, will become infected with CBSD. For clarity in visualising results, we have chosen to focus on five states that differ in cassava density and geographic location in Nigeria (Table 1). Detailed results for each state are included in the Supplementary Material. Overall, cassava density is highest is southern Nigeria and lowest in northern Nigeria (Fig 1A). The road network covers most of the country but is most dense in the south east region (Fig 1B).

**Table 1:** Summary of characteristics of the highlighted states.

State name	Cassava density	Average year infected	Identifier
Nasarawa	Low	2	L2
Plateau	Low	3	L3
Kebbi	Low	5	L5
Ogun	Medium	2	M2
Anambra	High	2	H2

## Results

The baseline survey strategy was originally developed for an endemic disease, cassava mosaic disease, and has not been optimised for CBSD nor for general epidemic disease detection. We assumed surveys would continue to select fields near roads and explored the impact of changing the baseline strategy by increasing the total number of fields surveyed and selecting fields non-uniformly across the country.

**Fig 2:**
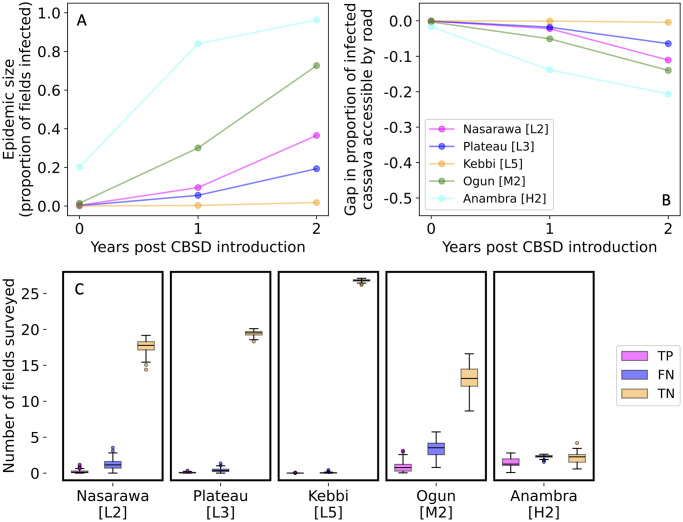
Early CBSD detection is limited by the number of fields surveyed per state. (A) Cumulative proportion of infected cassava fields in each of five selected states after CBSD is introduced. (B) The difference in the proportion of infected cassava fields within 1 km of a road and the total proportion of infected cassava fields in five selected states after CBSD is introduced. (C) For the three-year period after a state becomes infected, the average number of true positive, false negative, and true negative surveys per year. The box shows the 1^st^ to 3^rd^ quartile of the data with a line at the median value. The whiskers indicate 1.5x the inter-quartile range and any values outside of that range are shown as fliers. The times at which each state became infected from replicate simulations are shown in S3 Fig.

Initially, we explored the impact of limiting surveys to fields within 1 km of a road. Our results from model simulations indicate that the rate that CBSD infection bulks up in a state and the number of infected fields accessible to surveyors near the road varies amongst states (Fig 2A and 2B). However, in the first three years after CBSD has spread to a state, the gap between the total infected cassava and accessible infected cassava is relatively small, suggesting that this is not a limiting factor for early detection of CBSD (Fig 2B). Within the first three years after a state was first infected, the majority of fields surveyed in most states were true negatives (surveys conducted in fields without CBSD). Much smaller proportions were either false negatives (surveys conducted in fields infected with CBSD where the infection is missed) or true positives (surveys in infected fields infected with CBSD where the infection is detected) (Fig 2C). These results suggest that the main limitation is not the within-field survey strategy but the total number of fields surveyed per state because the number of fields where infection was missed was smaller than the number where no plants were infected. The gap between the number of false negatives and true negatives is smaller in Anambra state because the higher density of cassava plants in the state favoured more rapid spread of CBSD than in the other states. Therefore, we explored two different strategies for increasing the number of surveys per state: increasing the total number of fields surveyed annually and selecting fields nonuniformly for survey within Nigeria.

**Fig 3:**
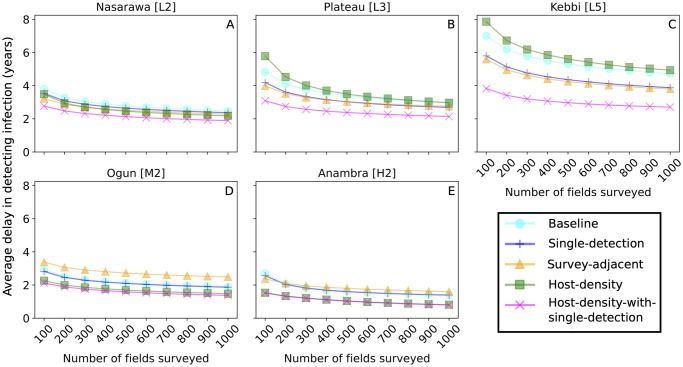
The average delay in detecting CBSD for different survey allocation strategies and number of fields surveyed annually. The y-axis is the average detection delay in years, and the x-axis is the total number of fields surveyed annually. Nasarawa is shown in (A), Kebbi is shown in (B) Plateau is shown in (C), Ogun is shown in (D), and Anambra is shown in (E).

As an initial example, Plateau, which is located in central Nigeria (Fig 1), has very low cassava density, and was infected on average five years after the simulated introduction of CBSD to Nigeria (Table 1). Regardless of the survey allocation method, increasing the number of fields surveyed per year decreased the lag between CBSD introduction and detection (Fig 3B). These incremental gains were larger when the number of fields surveyed increased from 100 to 200 annual surveys, with much smaller improvements when going from 900 to 1,000 surveys (Fig 3B). The ranking of the five survey allocation strategies was the same within Plateau, irrespective of the numbers of fields surveyed, suggesting that the allocation method was particularly important. Within this state, the *Host-density-with-single-detection* strategy performed notably better than the other strategies, to the extent that surveying 100 fields was as effective as surveying 1,000 fields with the next most effective survey allocation strategy, *Single-detection*.

Changing the total number of fields surveyed had a smaller impact than the survey allocation strategy in all the states (Fig 3). Within a single state, the rankings of the survey allocation strategies did not change as the number of annual surveys increased; however, the rankings did vary amongst states. Overall, *Host-density-with-single-detection* consistently detected CBSD sooner than the *Baseline* strategy, and *Host-density* outperformed the *Baseline* in states with medium or high-density cassava.

Evaluating the performance of different survey allocation strategies using delay in detection had limitations for comparing results across states because the delay metric is partially driven by the extent of road coverage of the states. One alternative is to compare the relative size of the epidemic at detection. Because the rate of epidemic spread tends to be slower in regions with lower cassava or whitefly density, comparing the epidemic size also takes into account the practical impact of delayed detection in a given state.

**Fig 4:**
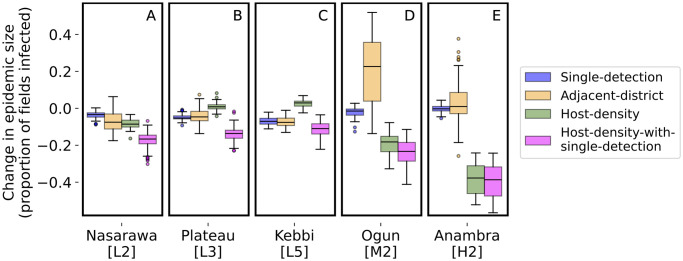
The change in epidemic size at detection when surveying 600 fields annually. For each subplot, the y-axis is the change in epidemic size for each of the alternative survey strategies compared with the baseline strategy, and the x-axis is labeled with the state name.

Next, we compared the size of the epidemic when CBSD was detected for each of the alternative strategies compared with the *Baseline* strategy when surveying 600 fields annually. Our results indicated that the *Host-density-with-single-detection* strategy detected CBSD when the epidemic was smaller compared with the *Baseline* and other strategies (Fig 4 and S2 Fig). The effectiveness of the *Host-density* strategy was however more variable. It was comparable to *Host-density-with-single-detection* in states with average to high cassava density (Fig 4D and 4E). However, in states with low cassava density, the performance was much worse. This was because without the additional constraint of not surveying fields in states where CBSD has been detected, a small number of fields would always be surveyed in states with low host density (Fig 4A–4C). The *Single-detection* survey strategy performed similarly to the *Baseline* strategy, and the *Survey-adjacent* strategy performed the same or worse than the *Baseline* strategy. It is likely that detection of CBSD within a given state lagged too far behind the disease front for concentrating surveys in the adjacent states to be helpful.

**Fig 5:**
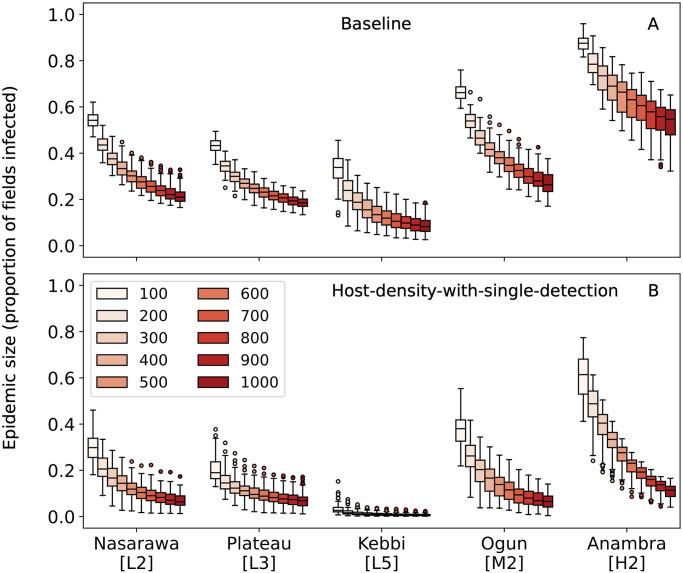
The change in epidemic size at detection relative to the number of fields surveyed annually. For each subplot, the y-axis is the proportion of cassava in a state infected at CBSD detection. (A) uses the *Baseline* survey allocation strategy and (B) uses the *Host-density-with-single-detection* strategy.

For the *Baseline* and *Host-density-with-single-detection* strategies, we compared how changing the number of fields surveyed annually impacted the epidemic size at detection. For any given number of annual fields surveyed, *Host-density-with-single-detection* outperformed the *Baseline* strategy in all states (Fig 5 and S3 Fig). Additionally, as when using detection lag as a metric, the incremental gains were larger when fewer fields were surveyed annually and in states with more dense cassava plants. This difference was particularly pronounced for Ogun and Anambra states because at high densities of cassava crops, CBSD spread quickly enough in simulations that small differences in average time to detection were amplified.

There are practical limitations to the number of annual surveys that can be conducted due to the availability of trained surveyors. One potential solution is to effectively increase the number of surveyors by using automated image classification algorithms to diagnose plants based on photographs of disease symptoms on a plant, thereby allowing people to conduct surveys for CBSD with minimal training. We ran simulations with more annual surveys for the *Baseline* and *Host-density-with-single-detection strategies*, assuming a lower per plant probability of CBSD detection, which is in line with the performance of current image classification algorithms.

**Fig 6:**
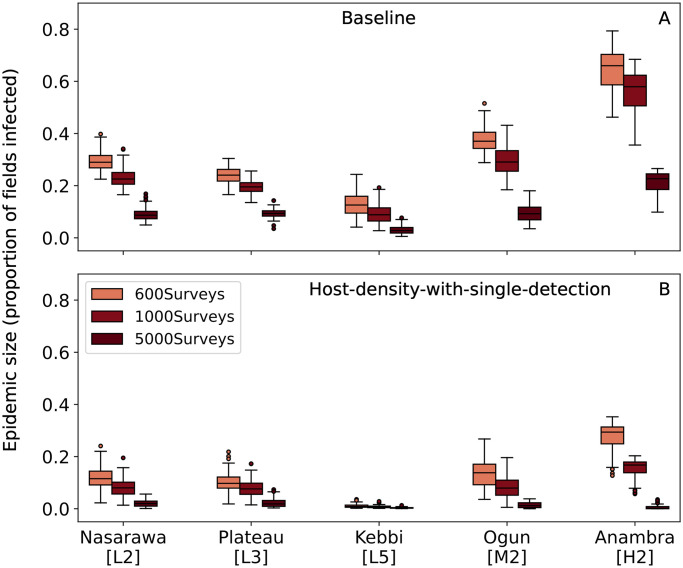
The change in epidemic size at detection when mass surveying fields. For each subplot, the y-axis is the proportion of fields with infected cassava in a state at CBSD detection. (A) uses the *Baseline* survey allocation strategy and (B) uses the *Host-density-with-single-detection-strategy*.

When surveying 5,000 fields annually, CBSD was detected at a smaller epidemic size for both the *Baseline* and *Host-density-with-single-detection* allocation strategies, and the gap in performance between the strategies was much closer although *Host-density-with-single-detection* was still superior (Fig 6). The results suggest that automated image classification could be a promising way to supplement expert surveys in the future, particularly when using the *Baseline* strategy, albiet the current analyses were carried out with an implausibly low false positive rate of zero.

Because the initial infection was seeded in different locations in Nigeria across all the simulated epidemics and due to model stochasticity, states became infected on average in different years in different simulations (S1 Fig). This between-run variance represents meaningful uncertainty in how CBSD might be introduced to Nigeria and then how the real epidemic would progress, and because only one epidemic trajectory would occur in real life, it is important for a survey allocation strategy to achieve some minimal benchmark of performance across a wide range of simulated epidemics. To quantify this, we used the benchmark criterion of detecting CBSD spread within three years of introduction to a state in at least 90% of field survey sub-samplings per epidemic trajectory. Then, for each of 100 independent epidemic trajectories, we could ask what minimum number of fields surveyed annually would be required to hit the benchmark using different survey allocation methods.

**Fig 7:**
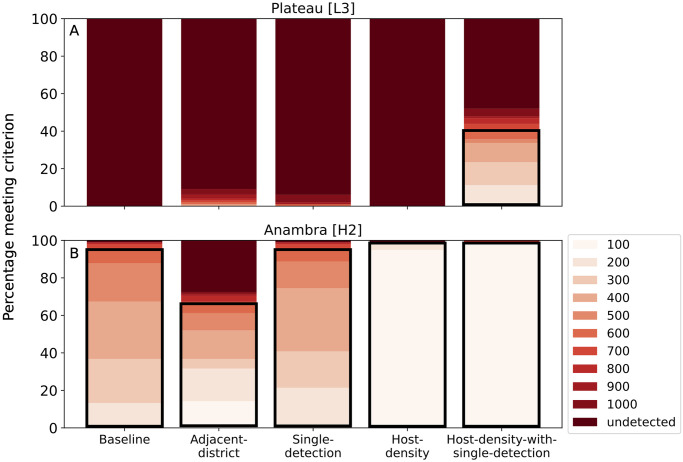
Host-density-with-single-detection consistently detects CBSD within three years when surveying a small number of fields. Stacked bar chart with boxes for the minimum number of surveys needed to hit the benchmark for the different epidemic trajectories. A black box is drawn around 600 or fewer surveys, to indicate expected performance with current number of surveys. Plateau is shown in (A) and Anambra is shown in (B).

In Plateau, it was difficult to detect CBSD spread quickly because of the low density of cassava plants, and even when surveying 1,000 fields annually, only the *Host-density-with-single-detection* strategy was consistently able to hit the benchmark (Fig 7A). In Anambra state, the higher cassava density made detection easier, and only the *Single-detection* strategy was unable to meet the benchmark consistently (Fig 7B). Additionally, for the *Host-density* and *Host-density-with-single-detection* strategies, it only required surveying 100 fields annually to have the same effectiveness as surveying 600 fields annually using the *Baseline* or *Survey-Adjacent* strategy.

## Discussion

We have shown that the number of fields surveyed in newly infected states is the limiting factor for detection of CBSD. Changing the survey allocation strategy to increase the number of fields surveyed in regions of the country at higher risk greatly increased the efficiency of detecting CBSD spread to new states. Increasing the total number of fields surveyed each year also added a small benefit. Specifically, the model indicated that surveying fields only in states where CBSD has not been detected and choosing field locations proportional to cassava density improves survey strategy performance for a set of key metrics associated with early detection. Hence, following the *Host-density-with-single-detection* strategy reduced the average time to detect infection in a new state, the size of the epidemic at first detection, and the total number of fields surveyed annually that would be needed to consistently detect epidemic spread in new states.

Previously, there have been concerns that limiting surveys to fields near roadways would bias detection of cassava mosaic disease; however, we find that for CBSD, only surveying fields within 1 km of a road did not meaningfully limit the total proportion of the infection epidemic that is available for surveillance in the early years after a state is infected (Fig 2B) [12]. The road network in Nigeria is fairly dense with 23% of the land area and over half of cassava fields accessible within 1 km of a road. With this level of accessibility, in the first few years after CBSD spreads to a state, the percentage of infected, road-accessible plants increases at a similar rate to overall epidemic spread (Fig 2A and 2B). However, in settings where less cassava is grown near the road network, it may be necessary to extend the buffer further away from the road for early detection of CBSD incursion.

Most surveyed fields were uninfected during the early stages of an epidemic and our results indicated low levels of false negatives (Fig 2C) using the current within-field survey protocol involving 30 plants. Hence, we conclude that optimisation of the within-field survey strategy is less important than optimising the country-level survey strategy to ensure as many fields as possible are conducted in newly infected states.

While we assume the road network is likely to be reasonably accurate and the majority (86%) of recorded past cassava surveys all lay within the current road buffer, a more pertinent question arises about the accuracy of host density across Nigeria [38]. For the cassava map, the host density is based on state level cassava production and harvested area data and then disaggregated to 1 km^2^ cells based on population estimates [35]. The availability of state-level cassava data for Nigeria supports the accuracy of the map, but it is difficult to estimate the error in the underlying population and production estimates. Ideally, the cassava maps would also include information on varietal distribution to help estimate the percentage of plants in a region that display foliar symptoms when infected [39–42]. Data on the prevalence of other cassava diseases or the impact of abiotic stress could also potentially help to estimate the likelihood of misdiagnosing foliar symptoms as CBSD by visual observation.

It is also worth noting that between countries, the size of administrative units and the scale at which a response is coordinated will vary. Because Nigeria is a federating republic, evaluating the performance of surveillance strategies at the state levels is sufficient. For countries where the size of the administrative unit is much larger or smaller than in Nigeria, the relative ranking of survey allocation strategies or the required number of annual surveys may change. Additionally, if the CBSD response is being coordinated at a national or transnational level, it might also be better to evaluate the surveillance strategy performance in ways that are independent from administrative units and to concentrate the surveys by regions instead of states. Importantly too, we focused in this paper on optimising surveillance strategies for early detection of disease arising from the direct introduction of CBSD through infected planting materials somewhere within Nigeria. If there were evidence for invasion of CBSD into Cameroon immediately to the east of Nigeria, there is likely to be prior warning for Nigeria and surveillance strategies would then be adapted to give greater emphasis to states adjoining Cameroon (Adamawa and Taraba states).

The *Baseline* surveillance strategy was developed for cassava mosaic, an endemic viral disease and as a result fields were selected approximately uniformly across the entire country for surveillance [43]. We find that the *Baseline* strategy can be improved upon for early detection of epidemics of CBSD within states in Nigeria, in particular by responding to the dynamics of disease, by limiting surveillance to states where the disease had not been detected and choosing fields to survey proportional to host density (Figs 3 and 4). The *Single-detection* strategy gave little added benefit over the *Baseline*, while the *Host-density* strategy only benefitted states with high cassava density (Figs 3 and 4). The *Host-density-with-single-detection* strategy detected CBSD epidemics sooner and when the epidemic was smaller in newly infected states. Moreover, by focusing on non-detected states and selecting sampling sites according to host density, the strategy consistently detected CBSD within three years of introduction in a state across all simulated disease trajectories and required surveying a much smaller number of fields each year to do so compared with other methods (Figs 3, 4 and 7).

It is widely known that survey precision increases if a region is stratified such that variation within strata is less than variation between strata with associated guidelines for optimal distribution of samples in stratified random sampling [43]. In an endemic setting, the geographic disease prevalence is likely to be relatively constant between years such that it may be possible to stratify regions according to expected disease prevalence. However, in epidemic scenarios, the year-to-year variation makes this type of stratification infeasible. Stratifying the density of fields surveyed according to cassava density is a way to take advantage of the fact that CBSD tends to spread at a faster rate in cassava dense areas. Dynamic stratification for crop disease surveillance for early detection of invading pests and pathogens merits further detailed analysis [11].

Surveying more fields per year increases the effectiveness of detecting CBSD (Figs 3 and 5) although the impact is smaller than changing survey allocation strategies (Figs 3 and 5). The modelling results indicate that the performance of the *Baseline* survey strategy and the *Host-density-with-single-detection* strategy starts to narrow only when 5,000 fields are surveyed each year (Fig 6). Although surveying fields in a subset of states may be sufficient for disease detection, in some cases there are separate reasons to continue surveying fields in endemic regions, such as looking for introduction of other cassava diseases, farmer education, or monitoring the effectiveness of management interventions [44–46]. In those cases, because of practical limitations, it is unlikely to be feasible to increase the number of fields surveyed annually to the point of conducting 5,000 per year. Using image classification algorithms to detect CBSD automatically has the potential to increase capacity by allowing extension workers and farmers to diagnose CBSD infected plants instead of relying solely on expert surveyors.

For ease in comparing different allocation strategies, we assumed that expert surveyors could identify CBSD with 100% efficiency. In practice, the true value is likely to be lower and dependent on geographic region, not the allocation method. Previous modelling suggested that the per plant true positive rate is less important for detecting the presence of CBSD in a field compared with the total number of plants surveyed or the survey timing [10]. Similarly, we found that decreasing the per plant true positive rate from 100% to 75% does not meaningfully change the size of the epidemic at detection (Figs 5 and 6). Field trials using the Nuru app for CBSD found that the per plant true positive rate using the app was 73% compared with 81% for expert surveyors [37, 47], suggesting that the current true positive rate would not be a limitation to using an app such as Nuru.

An important caveat to using Nuru, however, is that our analysis does not consider the false positive rate of misclassifying plants as being infected with CBSD. For Nuru specifically, the false positive rate ranges from 3% for plants with pronounced green mite damage up to 74% for plants with mild cassava mosaic disease symptoms [47]. Predicting the expected false positive rate for a region using image classification algorithms is difficult because it will depend on the cassava variety and other sources of biotic and abiotic stress. For initial detection of CBSD in West Africa, the false positive rate is likely to be a particular challenge because the high prevalence of cassava mosaic disease [19, 48–50]. Implementation of an approach like Nuru would likely require using a secondary confirmatory test such as checking for root necrosis or molecular validation, which may be logistically infeasible for the number of expected false positives [51, 52].

Early detection is key to providing information to policy makers for implementing successful control efforts [10, 11]. With the recent identification of CBSD resistant germplasm [53] and new models for sustainable clean seed systems being piloted in Nigeria and Tanzania [54], there are more potential strategies available to mitigate yield losses and an even greater need for modelling to help guide deployment and prioritisation of management strategies in the event of an outbreak. We find that a conventional approach illustrated by the *Baseline* strategy lags behind alternative strategies in detecting CBSD after the epidemic spreads into a new state (Fig 3). By concentrating the number of available surveys into states with higher cassava density and where CBSD has yet to be detected, the alternative strategies (most notably *Host-density-with-single-detection*) can be optimised to consistently detect spread of CBSD to new regions faster, when the epidemic is smaller, and to do so more consistently.

## Supporting information

S1 Fig Distribution of initial introduction time to each state across infection trajectories. (TIF)

S2 Fig The change in epidemic size at detection when surveying 600 fields annually for all states. For each subplot, the y-axis is the change in epidemic size for each of the alternative survey strategies compared with the baseline strategy, and the x-axis is labeled with the state name.
(TIF)

S3 Fig The change in epidemic size at detection relative to the number of fields surveyed annually. For each subplot, the y-axis is the proportion of cassava in a state infected at CBSD detection. (A, C, E) use the baseline survey allocation strategy and (B, D, F) use the Host-density-with-single-detection strategy.
(TIF)

S1 Checklist Inclusivity in global research. (DOCX)
