# Supplementary figures and images for: Computational models for improving surveillance for the early detection of direct introduction of cassava brown streak disease in Nigeria
Source: PLoS One. 2024 Aug 21;19(8):e0304656. doi: 10.1371/journal.pone.0304656 (PMC11338456; doi:10.1371/journal.pone.0304656)

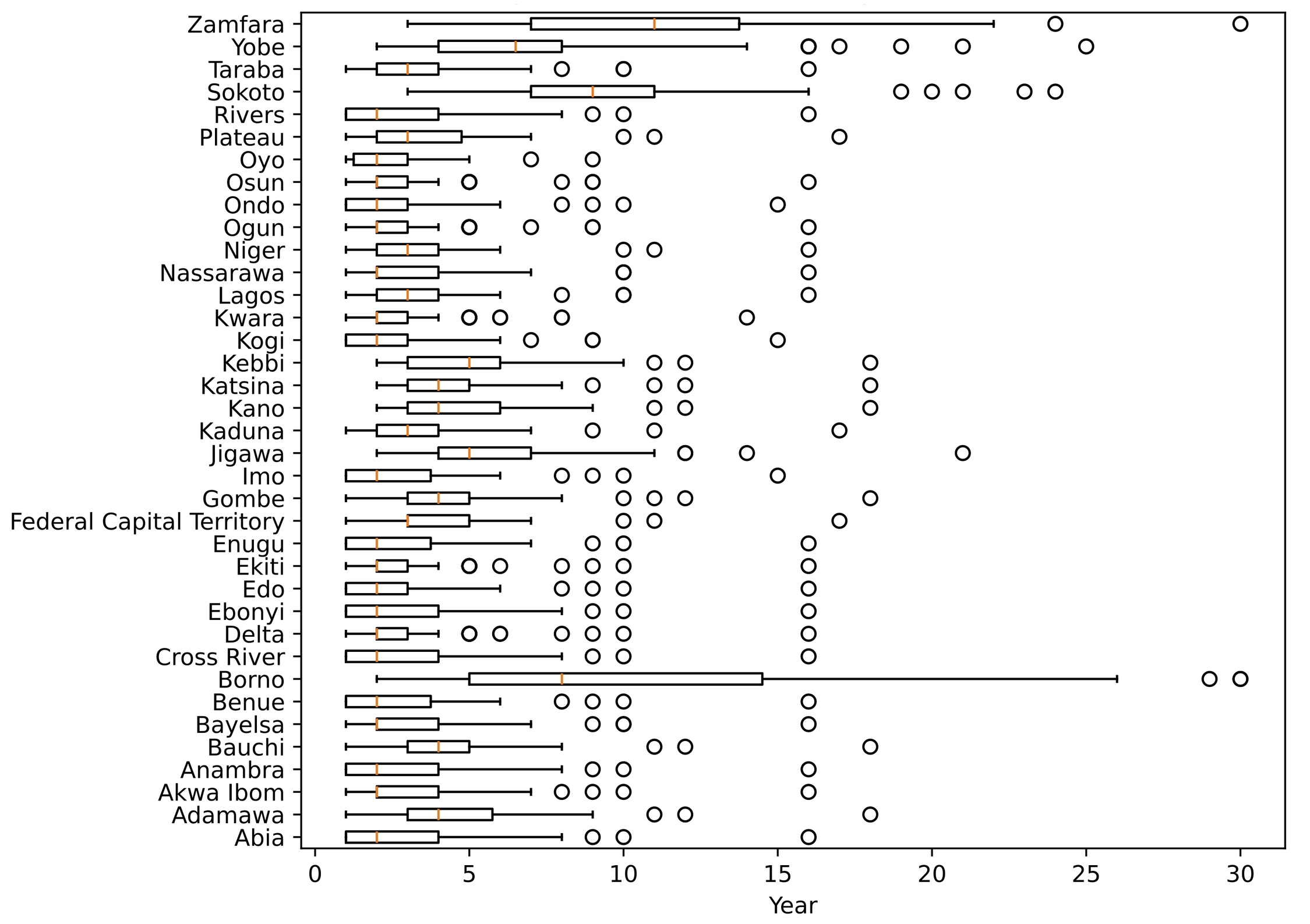

Supplement: S1 Fig — (TIF) [file pone.0304656.s001.tif]

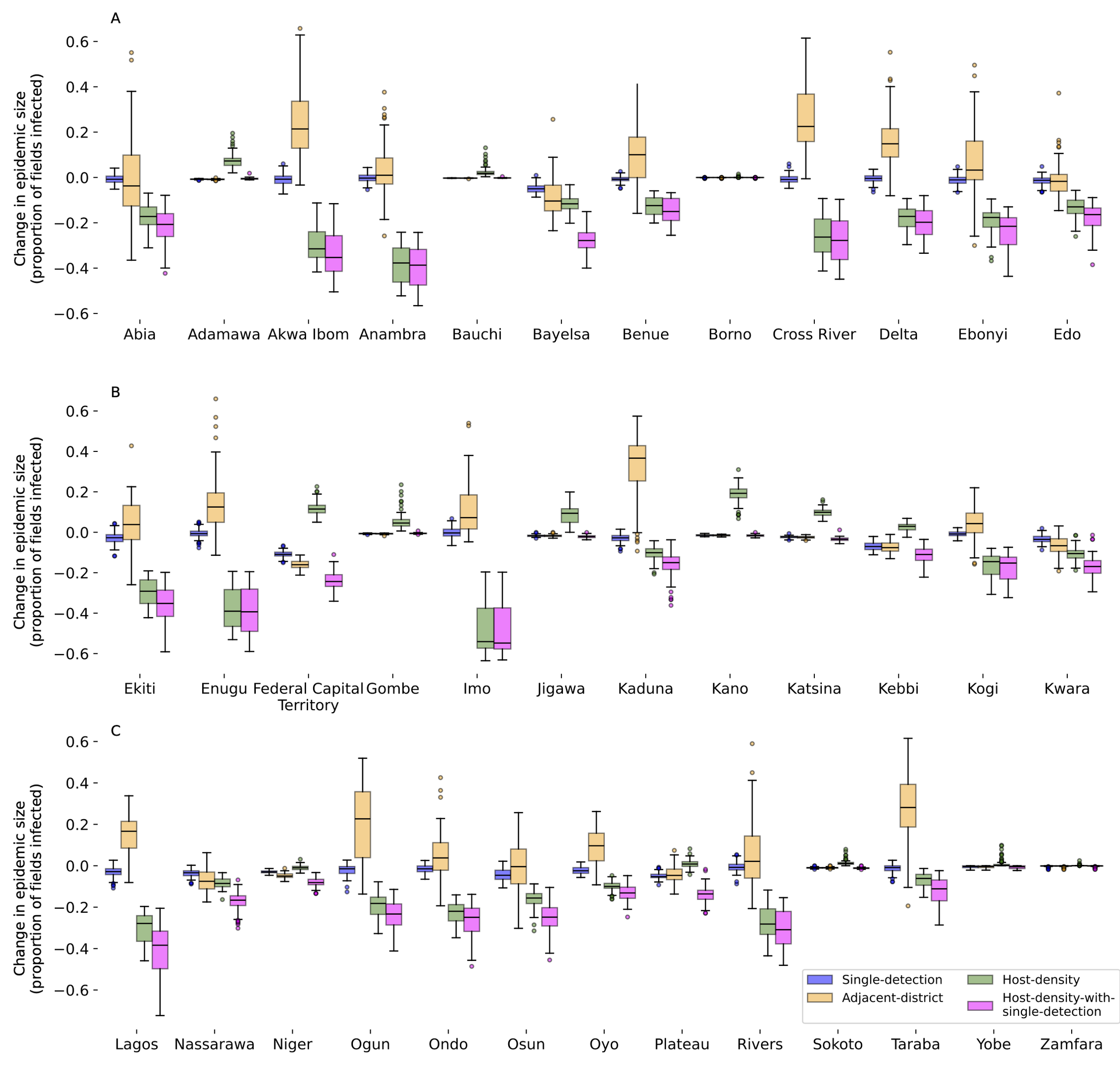

Supplement: S2 Fig — For each subplot, the y-axis is the change in epidemic size for each of the alternative survey strategies compared with the baseline strategy, and the x-axis is labeled with the state name. (TIF) [file pone.0304656.s002.tif]

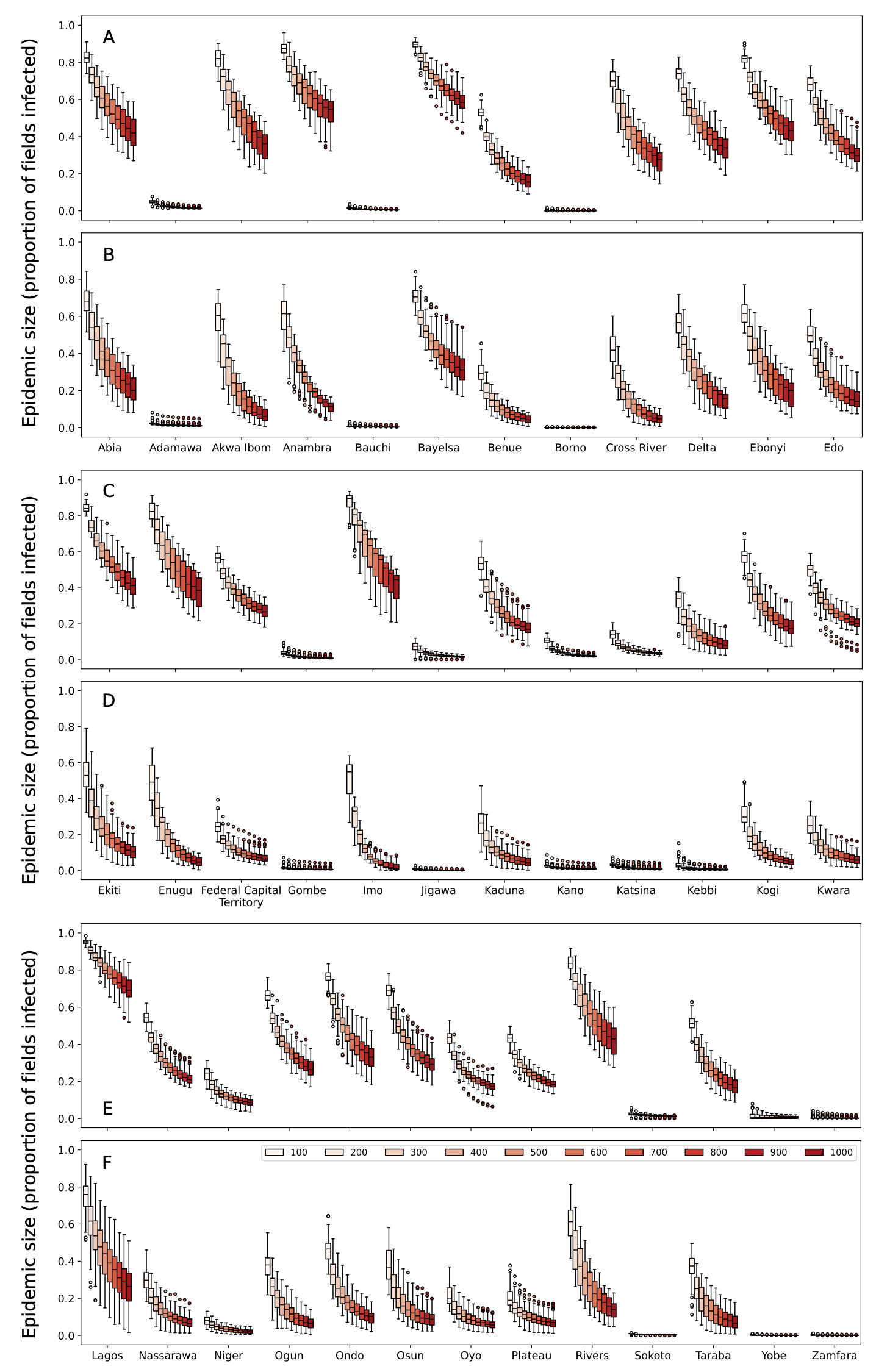

Supplement: S3 Fig — For each subplot, the y-axis is the proportion of cassava in a state infected at CBSD detection. (A, C, E) use the baseline survey allocation strategy and (B, D, F) use the Host-density-with-single-detection strategy. (TIF) [file pone.0304656.s003.tif]
